# Assembling carbon quantum dots to a layered carbon for high-density supercapacitor electrodes

**DOI:** 10.1038/srep19028

**Published:** 2016-01-12

**Authors:** Guanxiong Chen, Shuilin Wu, Liwei Hui, Yuan Zhao, Jianglin Ye, Ziqi Tan, Wencong Zeng, Zhuchen Tao, Lihua Yang, Yanwu Zhu

**Affiliations:** 1 Key Laboratory of Materials for Energy Conversion, Chinese Academy of Sciences, Department of Materials Science and Engineering, University of Science and Technology of China, 96 Jin Zhai Rd, Hefei, Anhui, 230026, P.R. China; 2 iChEM, University of Science and Technology of China, 96 Jin Zhai Rd, Hefei, Anhui, 230026, P.R. China

## Abstract

It is found that carbon quantum dots (CQDs) self-assemble to a layer structure at ice crystals-water interface with freeze- drying. Such layers interconnect with each other, forming a free-standing CQD assembly, which has an interlayer distance of about 0.366 nm, due to the existence of curved carbon rings other than hexagons in the assembly. CQDs are fabricated by rupturing C_60_ by KOH activation with a production yield of ~15 wt.%. The CQDs obtained have an average height of 1.14 nm and an average lateral size of 7.48 nm, and are highly soluble in water. By packaging annealed CQD assembly to high density (1.23 g cm^−3^) electrodes in supercapacitors, a high volumetric capacitance of 157.4 F cm^−3^ and a high areal capacitance of 0.66 F cm^−2^ (normalized to the loading area of electrodes) are demonstrated in 6 M KOH aqueous electrolyte with a good rate capability.

Supercapacitors (SCs), also known as ultracapacitors, electrostatically store charges on the surface of electrodes usually made from porous carbon, and thus have an energy density lower than that of batteries^1^. There has been a constant effort to improve the energy stored in SCs. According to the calculation of energy density (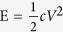), one way to improve the energy density is to increase the specific capacitance, given a certain operation voltage. In the past decade, much research has been done on pursuing novel electrode materials with high gravimetric capacitance such as by compositing carbon with high-capacitance materials^2^,^3^, by developing novel carbon materials such as graphene^4^, or high-surface-area porous carbons^5^,^6^. For most recently developed electrode materials with largely improved gravimetric capacitive performance, however, they performed relatively low volumetric capacitances due to the low packing densities and/or the low areal loading of electrode materials^7^. Electrode materials with a reasonably high packing density (e.g. higher than activated carbon which is typically 0.4 ~ 0.8 g cm^−3^)^8^ is critical for practical applications of novel electrode materials in practical SCs devices. Recently, improved apparent densities of SCs electrode materials have been achieved with, e.g. chemically converted graphene (CCG) hydrogel films with a density of 1.33 g cm^−3^ made by vacuum filtrating hydrazine reduced graphene oxide^9^,^10^ or high density porous graphene macroform (HPGM) with a density of 1.58 g cm^−3^ made by hydrothermal treating and vacuum drying graphene oxide suspension^11^. As a result, the volumetric performance of carbon-based supercapacitors has been improved^12^,^13^,^14^.

Recently another type of carbon, named carbon quantum dots (CQDs), have attracted much attention due to their advantages in excellent aqueous solubility, robust chemical inertia, easy functionalization, low toxicity and strong photoluminescence^15^,^16^,^17^,^18^,^19^,^20^. Many applications have emerged referring to CQDs, such as use as photocatalysts^21^, in organic photovoltaic devices^22^, for bioimaging^23^, and in sensors^24^. On the other hand, to control over the optical and electronic coupling between individual CQDs, they are often to be assembled in a geometrically well-defined structure. For example, assembling graphene quantum dots (GQDs) made by electrochemical oxidation of graphene to nanotubes with anodic aluminum oxide membrane has been reported as a platform for Raman enhancement^25^. By assembling GQDs made by solvothermal method from graphite oxide on interdigital finger electrodes through electrophoretic deposition, a micro-supercapacitor has been fabricated demonstrating a specific capacitance of 534.7 μF cm^−2^ (to areal loading of electrodes) and a rate capability of up to 1000 Vs^−1^, simultaneously with an excellent power response and cycling stability^26^. Among all of these applications of CQDs, however, there are few reports on directly packing CQD powders to bulk electrodes of SCs, which is highly desired for CQD-based energy storage devices with potentially improved volumetric performance.

To fabricate CQDs a few methods have been developed, e.g. by purifying soot produced in the arc discharge synthesis of single-walled carbon nanotubes^27^, by laser ablation of carbon target^28^,^29^, by oxygen plasma treatment of single-layer graphene^30^, or by catalytically opening C_60_ cages on ruthenium^31^. Electrochemically oxidizing graphite rods^32^,^33^, hydrothermally cutting graphene sheets^34^, acidic treatment of carbon fibers^23^, or microwave pyrolysis of polyethylene glycol 200 and saccharide^35^,^36^ have also been utilized to obtain CQDs. Recently, various Hummers’ methods for rupturing C_60_ molecules to CQDs have been developed^37^,^38^. On the other hand, KOH activation is one widely used method to create high porosity^39^, *e*.*g*. in the processing of carbon precursors (cokes, coals, etc.) for the preparation of activated carbons^40^,^41^. Studies on the synthesis of activated carbons suggested that KOH activation of carbon generally follows the reaction 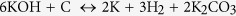, and the sequential decomposition of 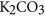 and/or reactions of K/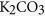/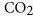 with carbon^42^. KOH activation has also shown effective in processing sp^2^ bonded carbons (e.g. carbon nanotubes (CNTs)^43^, carbon nanofibers (CNFs)^44^, graphene^5^, fullerene) for enhancement in performance of SCs. KOH activated microwave exfoliated graphite oxide, known as aMEGO^5^, has a remarkable Brunauer-Emmett-Teller (BET) specific surface area (SSA) of up to 3100 m^2 ^g^−1^. Fullerene (C_60_) has also been activated by KOH to form a novel three-dimensional (3D) porous carbon architecture, named a-C_60_, in our group recently. As far as we know, there is no report on using KOH activation to process fullerenes for the synthesis of CQDs.

In this work, CQDs are fabricated through reacting C_60_ molecules with excess KOH at elevated temperatures, and are assembled to a layered structure for being used as electrode materials in SCs. The absolute quantum yield (QY) of as-prepared CQD suspension is 7.4% when being excited by light with a wavelength of 350 nm, measured by integrating sphere technique; thus as-prepared CQD suspension demonstrates useful applications in bioimaging of HepG2 cells. Upon freeze drying, CQDs self-assemble to form a stacking of quasi-two-dimensional layers. The production yield of CQD layered structure reaches 15 wt.% from raw C_60_ precursor. After annealing, the SC electrodes made from CQD assembly has a density of up to 1.23 g cm^−3^ and demonstrate a volumetric capacitance of 157.4 F cm^−3^ and an areal capacitance of 0.66 F cm^−2^ (normalized to the loading area of electrodes) measured in 6M KOH electrolyte at a current density of 0.5 A g^−1^.

## Results

### Characterizations of CQDs fabricated from C_60_ by KOH activation

For the synthesis of CQDs, 300 mg of C_60_ powder was mixed with 9 g KOH in a Ni crucible at room temperature. The mixture was melted in a tube furnace at 400 ^o^C for about 3 min and was taken out for stirring, followed by cooling to room temperature. Then the mixture in Ni crucible was put into tube furnace again and heated at 600 ^o^C for 6–8 min at Ar ambience. The reaction was terminated by quickly pulling out and cooling the Ni crucible to room temperature. Bubbles were observed and gaseous species were released during the heating process. The product in the Ni crucible was washed by de-ionized (DI) water and filtered to remove big particles. The suspension through filtration was dialyzed in DI water for 6 days to obtain a clear final suspension sample.

**Figure 1:**
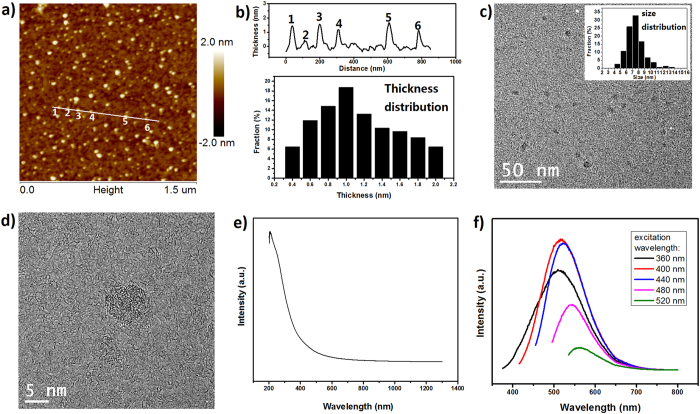
(**a**) AFM height figure. (**b**) (Up panel) Line section of white line in (**a**). Heights of 6 CQDs units are ~1.45, ~0.38, ~1.56, ~1.17, ~1.67 and ~1.12 nm, respectively. (Lower panel) Height distribution of CQDs counted from (**a**). (**c**) TEM of CQDs and inset shows size distribution. (**d**) HR TEM of an isolated CQD. (**e**) UV-vis-NIR absorption of CQD suspension. (**f**) Photoluminescence spectra of CQD suspension excited by light with various wavelengths.

The morphology of CQDs in suspension has been investigated with atomic force microscopy (AFM) by casting CQD suspension with a concentration of 0.05 mg ml^−1^ on a mica substrate, followed by drying in air. The typical topological AFM image is shown in Fig. 1a. A line taken from the image indicates that the height of randomly selected 6 individual CQDs falls between ~0.38 and ~1.67 nm. A statistics based on 310 CQDs from AFM image suggests that the height distribution of the CQDs centers at ~1 nm, as shown in the lower panel of Fig. 1b. The average height is ~1.14 nm, close to the typical value documented for graphene oxide platelets^45^,^46^. Transmission electron microscopy (TEM) images are shown in Fig. 1c and Figures S1a,b, from which CQDs with a lateral size of less than 10 nm can be clearly distinguished. The CQDs have a typical size of 7–8 nm from measurements of 171 CQDs. The high resolution TEM (HRTEM) image shown in Fig. 1d further indicates the CQDs have an amorphous microstructure. The reason that the typical size of CQDs is bigger than the diameter of C_60_ molecules is presumably thought to be related to the dangling-bonds created by KOH activation, which may lead to merging or assembly of C_60_ fragments^5^. However, CQDs did not form a 3D porous carbon architecture like in the cases of aMEGO and a-C_60_^5^, which can be explained by the very high ratio of KOH to carbon during the activation (e.g., the KOH/C_60_ ratio is 30, compared to a typical KOH/carbon ratio of 6–10 used in the preparation of aMEGO or a-C_60_) and a short activation duration (6–8 min compared to more than 1 h in the preparation of aMEGO or a-C_60_). Under such conditions the ration between C_60_ and KOH is more complete and C_60_ fragments have less opportunity to meet each other for possible restructuring. The curvature caused by the pentagons in CQDs and/or the functional groups introduced during the activation may explain the average height of more than 1 nm and the absence of crystalline structure as observed in graphene (Fig. 1d, Figures S1c,d).

As-prepared CQDs were characterized with UV-vis-NIR absorption spectroscopy and the spectrum obtained from CQD suspension (0.2 mg ml^−1^) is shown in Fig. 1e. Two peaks at ~206 nm and ~260 nm, are attributed to π-π* transition of aromatic C = C bonds and n-π* transition of C = O bonds, respectively^47^. Photoluminescence (PL) emission studies of as-prepared CQD suspension (0.2 mg ml^−1^) demonstrate an excitation-dependent PL (Fig. 1f), which was also observed from other CQDs^48^ and GQDs^49^. The emission peak shifts from 510 nm to 560 nm, when the excitation wavelength changing from 360 nm to 520 nm. Such an excitation-dependent PL is related to the size distribution of CQDs and/or to the distribution of different emissive site in CQDs^48^,^50^. As we simulated in previous work, the band gap of CQDs made by Hummers’ rupture of C_60_ molecules depends on the size of the CQDs^51^. On the other hand, the presence of small sp^2^ clusters isolated within the sp^3^ C-O matrix induced by oxygen-containing groups may lead to a localization of electron-hole pairs, facilitating radiative recombination especially for CQDs with large size^52^. Surface states introduced by oxygen-containing groups or other defects may also dominate the emission in some cases^18^. Absolute quantum yield of as-prepared CQDs, measured by integrating sphere technology, is 7.4% under the excitation of 350 nm. With bright PL and low cytotoxicity, CQDs have shown useful in bioimaging^23^,^53^. Figure S2 shows fluorescent images excited by 405 nm light, obtained by a confocal laser scanning microscope (CLSM) of HepG2 cells, one kind of mammalian cells, treated by as-prepared CQDs. As can be seen, most HepG 2 cells are blueish, which can be explained by the fact that small CQDs go into the cells more easily than big ones, thought as-prepared CQDs emit light covering from blue to yellow when being excited by 400 nm light shown in Fig. 1f.

### Assembling CQDs to a layered carbon

**Figure 2:**
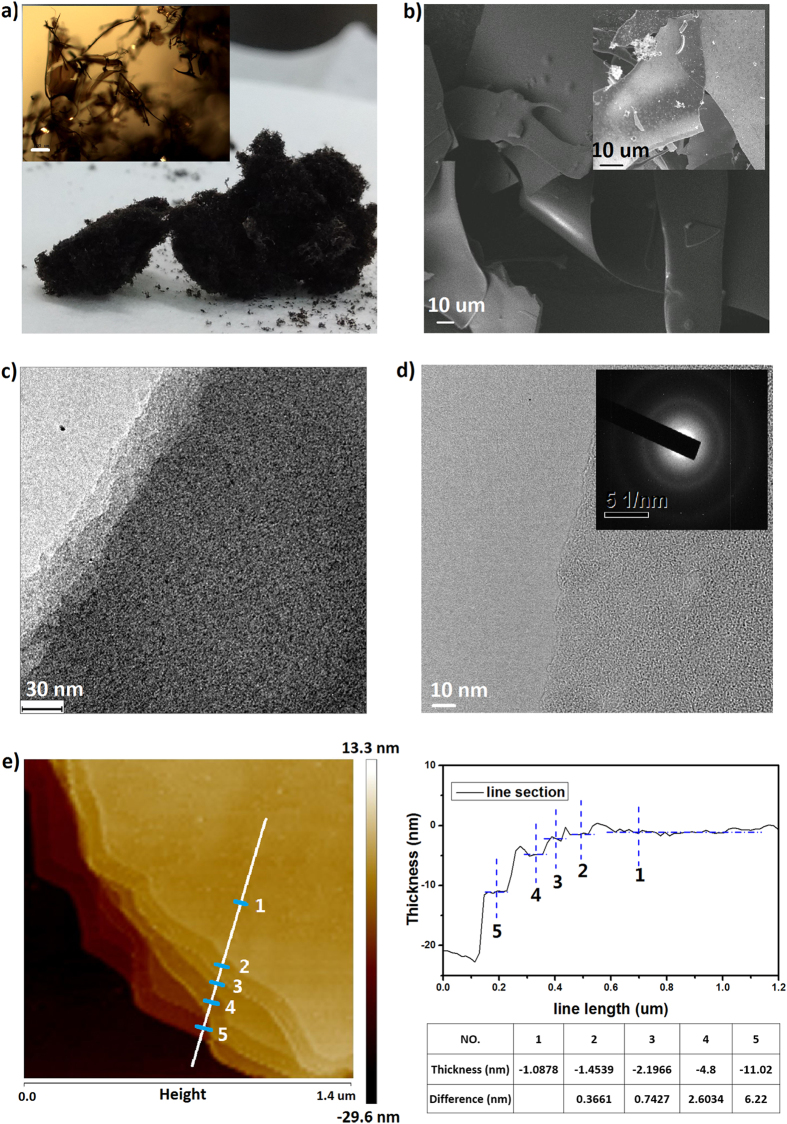
(**a**) Optical image of CQD assembly powder. (Inset) Optical image of sponge-like CQD_s-800_ taken by an optical microscope. The scale bar is 100 μm. (**b**) SEM of CQD_s-800_. (Inset) SEM of CQDs_-800_. TEM of (**c**) multilayer and (**d**) single layer of CQDs_-800_. (**e**) AFM of CQDs_-800_. Right panel shows line section of white line in the AFM image and the height collection at position marked by 1, 2, 3, 4 and 5 on the line.

CQD suspension in water was dried by freeze drying and the yield of dry product was about 15 wt.% from raw C_60_ precursor. Before freeze drying, the CQD suspension was firstly concentrated to a concentration of ~3 mg ml^−1^ and then freezed in a fridge for overnight. A free-standing sponge-like material was obtained from freeze drying of the pre-freezed CQD suspention, as shown in the optical image in Fig. 2a. An optical microscope image (inset of Fig. 2a) indicates that the CQD sponge consists of interconnected sheet-like assmbly of CQDs. Scanning electron microscopy (SEM) image shown in Fig. 2b demonstrates curved sheets with a size of from dozens of to hundreds of micrometers. It is worth noting that such as-prepared CQD asembly from freeze-drying can be readily redispersed in water without any processing such as ultrosonic or stirring. Thus annealing at 800 or 600 ^o^C for 30 min has been carried out to stablize the assembly and the annealed assembly is denoted as CQDs_-800_ after thermal treatment at 800 ^o^C or CQDs_-600_ after thermal treatment at 600 ^o^C. As shown in the SEM image in the inset of Fig. 2b, the assembly annealed at 800 ^o^C maintains the sheet-like morphology. By casting the water suspension of CQDs_-800_ on Cu grids, TEM images were taken to investigate the microstructure of annealed CQD assembly. From the TEM image in Fig. 2c, one can clearly distinguish a layer structure of the annealed assembly with well-defined contrast caused by the different numbers of layers at the edge. The TEM image in Fig. 2d shows a single layer of CQDs_-800_, with a morphology similar to graphene membrane but absent of hexagonal lattice fringes. The electron diffraction (inset of Fig. 2d) suggests that the layer structure has a polycrystalline feature, with a dominate diffraction ring corresponding to a lattice constant of about 0.219 nm. Such a lattice parameter is consistent with the (100) lattice space of GQDs^54^. Figure 2e shows the atomic force microscopy (AFM) image performed on CQDs_-800_. The vertical distances of the layers, measured between adjacent marks from top to down, are 0.366, 0.742, 2.603 and 6.220 nm, which all are roughly integral times of 0.366 nm; thus the interlayer distance in annealed CQD assembly can be assigned as about 0.366 nm, slightly larger than that of graphite. This is presumably considered to be related to curved carbon rings other than hexagons in the CQD assembly obtained by reacting C60 with KOH.

### Composition and SSA analysis

**Figure 3:**
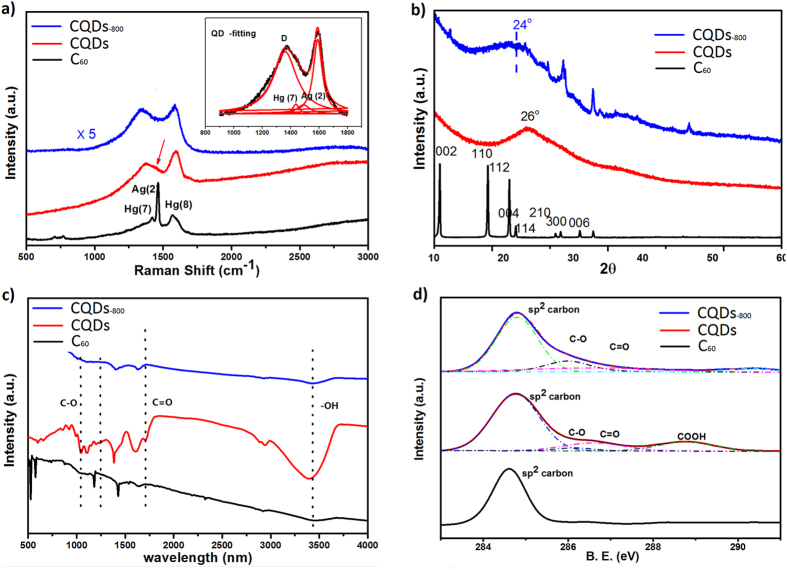
(**a**) Raman spectra, (**b**) XRD, (**c**) FT-IR, and (**d**) C 1s XPS spectra of C_60_, as-prepared CQD assembly and CQDs_-800_.

Typical Raman spectra of as-purchased C_60_, as-freeze-dried CQD asembly and CQDs_-800_ are shown in Fig. 3a. As we can see, the breathing modes of C_60_ cage located at lower wave numbers in the spectrum totally disappear after the C_60_ molecules are converted to CQDs, indicating that the cage of C_60_ molecules has been broken into fragments by KOH activation^55^. The vibration modes of C_60_ referring to pentagon shear (Hg (7), ~1428 cm^−1^), pentagon pinch (Ag (2), ~1469 cm^−1^) and hexagon shear (Hg (8), ~1570 cm^−1^)^56^,^57^ are broadened to two bands. With Lorentz fitting of the bands (Fig. 3a, inset), Hg (7), Ag (2) and Hg (8) can be distinguished. Even after annealing at 800 ^o^C, Hg (7) and Ag (2) still exist in the spectrum of CQDs_-800_, suggesting that pentagon and hexagon rings are still preserved in CQDs^55^. The strong band at ~1360 cm^−1^ corresponds to the defects-related D mode in graphitic carbon^58^. X-ray diffraction (XRD) in Fig. 3b also shows that the characteristic peaks of bulk C_60_ disappears after the activation processing. Upon freeze-drying, the XRD of CQD asembly shows a broad peak centered at ~26^o^, indicating the formation of a layered structure. After annealing at 800 ^o^C, the peak shifts to ~24^o^ and becomes broader. The interlayer distance calculated is about 0.37 nm, highly consistent with the AFM measurement. As discussed below, as-prepared CQDs include plenty of oxygen-containing groups and removing such groups by annealing may release gaseous species and create defects, leading to peak broadening. The production of gaseous species may blow and further open layers, explaining the peak shift to lower degree. The sharp peaks at 32.6^o^, 37.5^o^ and 54.1^o^ are indexed as K_2_O impurities.

Fourier transform infrared spectroscopy (FTIR) spectra shown in Fig. 3c indicate that the as-prepared CQD asembly contains oxygen-containing groups, such as C-O (alkoxy, stretching at ~1050 cm^−1^), C-O (epoxide/ether, stretching at ~1230 cm^−1^), C = O (carboxyl/carbonyl, stretching at ~1720 cm^−1^), -OH (hydroxyl, stretching at ~3410 cm^–1^)^59^. The carboxyl groups located at the edges allow CQDs to have negative charges due to the ionization of –COOH, promoting the excellent dispersion of CQDs in water^60^,^61^. After annealing at 800 ^o^C, the FTIR intensities of all oxygen-containing groups, especially those of carboxyl and hydroxyl groups, show a remarkable decrease. The C1s X-ray photoelectron spectroscopy (XPS) spectrum in Fig. 3d further shows four main carbon bonding types, i.e., C-C (~284.78 eV), C-O (~286.1 eV), C = O (~286.6 eV) and -COOH (~288.8 eV) from as-prepared CQD asembly^23^. The C/O atomic ratio estimated from XPS is 2.15. After annealing, C1s XPS spectrum shows that the proportion of sp^3^ carbon (C-O, C = O and -COOH) in the whole carbon is reduced from 36% to 28%; correspondingly the C/O atomic ratio in CQDs_-800_ is increased to 3.14. From O1s XPS spectra shown in Figure S3, the proportion of C = O in the whole oxygen-containing groups decreases from 51.6% to 20.4% after annealing.

**Figure 4:**
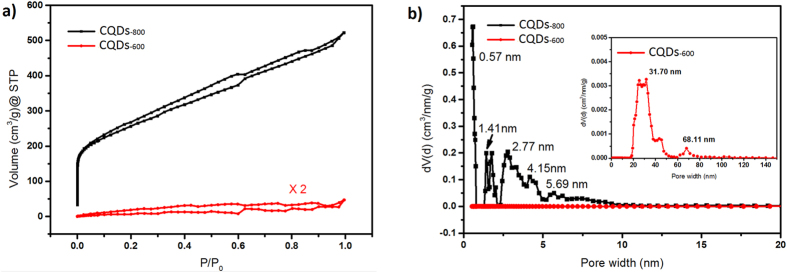
(**a**) Nitrogen adsorption isotherms of CQDs_-800_ and CQDs_-600_. (**b**) Pore size distributions of CQDs_-800_ and CQDs_-600_, obtained by DFT analysis of nitrogen adsorption isotherms. (Inset) Zoom in of pore size distribution of CQDs_-600_.

Nitrogen adsorption/desorption isotherms of CQDs are shown in Fig. 4a. Due to severe reduction occurring in the degas process, it is difficult to measure the adsorption characteristic of as-prepared CQD asembly. Thus CQDs_-800_ and CQDs_-600_ are compared to detect the change of porosity caused by annealing. The isotherms, as shown in Fig. 4a, reveal micropore filling in low pressure region for CQDs_-800_, and H1 hysteresis which refers to slit pores according to the International Union of Pure and Applied Chemistry (IUPAS) classification^62^. The BET SSA of CQDs_-800_ is calculated as 857.7 m^2 ^g^−1^, much higher than 6.9 m^2 ^g^−1^ obtained from CQDs_-600_. At the same time, the total pore volume is 0.733 cm^3 ^g^−1^ for CQDs_-800_ while it is only 0.028 cm^3 ^g^−1^ for CQDs_-600_. Pore size analysis of isotherms with quenched solid density functional theory (QSDFT) based on a slit-pore model, shown in Fig. 4b, indicates that the typical pore diameter in CQDs_-800_ is less than 5 nm while large pores dominate in CQDs_-600_. Detailed analysis shows that in CQDs_-800_ pores with typical size of 0.5 ~ 1 nm, 1 ~ 2 nm, 2 ~ 5 nm or larger than 5 nm contribute to pore volumes of 0.177, 0.083, 0.309, 0.163 cm^3 ^g^−1^, respectively.

## Discussion

Clearly, a novel carbon with layered yet porous strucutre has been obtained by treating C_60_ with KOH, followed by freeze-drying and annealing. In freeze-drying it is believed that ice crystals act as templates and the self-assembly occurs at ice crystals-water interface, as observed in the asembly of grpahene oxide (GO) under similar conditions^63^. The ice crystals could be formed during the pre-freezing of CQD suspension, leading to phase separation and aggregtion of CQDs in the frozen suspension. During freeze-drying, oxygen-contaning groups such as –OH and –COOH groups on CQDs, π-π interaction and dipole-dipole interaction between carbon layers and functionalized groups may benefit bonding and stacking of CQDs^64^,^65^,^66^. However, the existance of oxygen-containing groups make the stacking unstable, and thus as-prepared CQD asembly can be readily redispersed in water. The results of nitrogen adsorption/desorption isotherms analysis and XRD analysis above indicate further annealing removes the oxygen-containing groups and possibly some carbon atoms as well; the release of gaseous species and more defects created during annealing may contribute to the development of porosity and broadened interlayered distance in CQDs_-800_.

**Figure 5:**
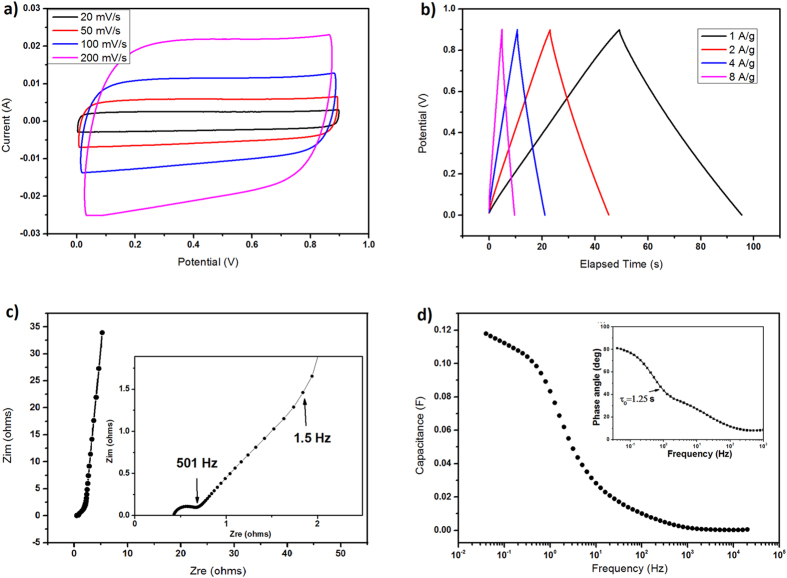
Electrochemical performance of CQDs_-800_. (**a**) Cyclic voltammetry (CV). (**b**) Galvanostatic charge/discharge curves. (**c**) Nyquist plot. (Inset) Magnified curve in the high-frequency range. (**d**) Frequency response of the capacitance estimated with a RC model. (Inset) Bode phase plot.

With the best practice for assembly of electrode materials in SCs^67^, performance of CQDs_-800_ and CQDs_-600_ was evaluated in a two-electrode symmetrical setup using 6 M KOH as electrolyte. Electrodes were prepared by combining 85 wt.% CQDs_-800_ or CQDs_-600_, 10 wt.% carbon black as conductive additive and 5 wt.% PTFE as adhesive. Such electrode membranes have a typical density of 1.08 g cm^−3^ and 1.23 g cm^−3^ for CQDs_-800_ and CQDs_-600_, respectively. Figure 5 shows the measurement of CQDs_-800_ electrodes in SCs. As shown in Fig. 5a, cyclic voltammetry (CV) testing demonstrates rectangular shapes from 0 to 0.9 V at various scan rates. The galvanostatic charge/discharge curves in Fig. 5b also show nearly ideal charge/discharge behaviors at various current densities. The specific capacitance, as calculated from the discharge curve at 0.5 A g^−1^, is 106 F g^−1^ (114.7 F cm^−3^ or 0.88 F cm^−2^ when normalized to the volume or loading area of electrode membranes, respectively). At a higher current density of 8 A g^−1^, the specific capacitance remains 84.4 F g^−1^ (91.2 F cm^−3^ or 0.7 F cm^−2^). The Nyquist plot shown in Fig. 5c, measured in a frequency range of from 1 MHz to 0.01 Hz, further indicates an excellent capacitive behavior of the carbon. From the magnified curve in the high-frequency range (inset of Fig. 5c), a transition between the resistance capacitance (RC) semicircle and the migration of electrolyte was observed at a frequency of about 501 Hz, corresponding to a resistance of 0.682 ohms. The diffusion of electrolyte ions stops at about 1.58 Hz^68^. The result is consistent with analysis of frequency response (Fig. 5d), which is based on a series RC model. The capacitance decreases sharply from ~0.12 F at about 3 Hz and remains 0.028 F at 10 Hz. The time constant τ_0_ in the Bode phase plot is 1.25 s (inset of Fig. 5d), indicating a superior frequency response of CQDs_-800_. In comparison, CQDs_-600_ does not show very rectangular CV curves (Figure S4), suggesting that pseudocapacitance is more obvious in the case of CQDs_-600_^69^, while the capacitance of CQDs_-800_ is mainly attributed to electrical double layer capacitance (EDLC). With higher density, however, SCs constructed from CQDs_-600_ have a higher volumetric capacitance of 157.4 F cm^−3^ (this value is in the medium level of state-of-the-art data in the literatures, see Table S1) and an areal capacitance of 0.66 F cm^−2^ at current density of 2 A g^−1^. After 4000 charge/discharge cycles at 2 A g^−1^, the volumetric capacitance CQDs_-600_ is 137.8 F cm^−3^, a retention of 87.5% (Figure S4). When being normalized to the BET SSA, the specific capacitance of CQDs_-600_ is as high as 1.85 mF cm^−2^, much higher than 12.3 μF cm^−2^ of CQDs_-800_ measured at 0.5 A/g or 10–70 μF cm^−2^ for graphitic carbon^70^. It is very likely that the oxygen-containing groups is dominant for the capacitive performance in CQDs_-600_^11^, while thermal treatment at 800 ^o^C removes more oxygen groups and results in an increase of SSA by developing more pores, leading to a higher contribution from EDLC CQDs_-800_.

In summary, we have developed a novel carbon by utilizing KOH activation to treat C_60_ followed by assembly with freeze-drying and annealing. As-prepared CQDs consist of abundant oxygen-containing groups and can be easily dispersed in water, for strong PL and potential applications in bioimaging of, e.g. HepG2 cells. Upon free-drying and annealing, CQDs assembled to a layered yet porous strucutre, demonstrating desired density and porosity in SC electrodes. Thus the carbon has shown superior volumetric capacitance and a good rate capability. The best volumetric capacitance is 157.4 F cm^−3^ in 6 M KOH electrolyte. With a high yield of 15 wt.%, the CQDs prepared and their assmebly could be used in future optoelectric, imaging or energy storage devices.

## Methods

### Materials

C_60_ powder was purchased from Suzhou Dade Carbon Nanotechnology Co., Ltd. The purity of C_60_ molecules is 99.9% by company’s data sheet. KOH (AR) was purchased from Sinopharm Chemical Reagent Co., Ltd.

### Preparation of CQDS from C_60_

Firstly, at room temperature 300 mg of C_60_ powder was mixed with 9 g KOH in a Ni crucible. Then the Ni crucible was put into a tube furnace at 673 K for about 3 min and the mixture in the crucible was melted. The crucible was taken out from the furnace and stirred, following by cooling to room temperature. Then the Ni crucible was put into a tube furnace and reacted for 6 ~ 8 min at 873 K, surrounding Ar ambience. During the reaction processing, bubbles were created and gaseous species were released, which may be H_2_ or CO_2_. Once the reaction time is up, the Ni crucible was pulled to room temperature zone to quickly cool down and terminate the reaction. After the reaction, the solid product was washed by DI water and filtered to remove big particles. Suspension product was gotten. To remove impurities, the suspension was then purified by dialysis in DI water for 7 days, and the specification of the dialysis-membrane was 500 ~ 5000 amu. Finally, a clear water suspension in light yellow color was obtained. To get carbon quantum dots (CQDs) assembly powder, the water suspension was frozen in a fridge firstly and then dried in a freeze-drying machine.

## Additional Information

**How to cite this article**: Chen, G. *et al.* Assembling carbon quantum dots to a layered carbon for high-density supercapacitor electrodes. *Sci. Rep.* **6**, 19028; doi: 10.1038/srep19028 (2016).

## Supplementary Material

Supplementary Information
