# Supplementary material for: Assembling carbon quantum dots to a layered carbon for high-density supercapacitor electrodes
Source: Sci Rep. 2016 Jan 12;6:19028. doi: 10.1038/srep19028 (PMC4709517; doi:10.1038/srep19028)
Supplement: Supplementary Information [file srep19028-s1.pdf]

Supporting Information

Assembling carbon quantum dots to a layered carbon for high-density supercapacitor electrodes

Guanxiong Chen[1], *Shuilin Wu*[1], *Liwei Hui*[1], *Yuan Zhao*[1], *Jianglin Ye*[1], *Ziqi Tan*[1], *Wencong Zeng*[1], *Zhuchen Tao*[1], *Lihua Yang*[1], *and Yanwu Zhu*[1,2]

[1]Key Laboratory of Materials for Energy Conversion, Chinese Academy of Sciences, Department of Materials Science and Engineering, University of Science and Technology of China, 96 Jin Zhai Rd, Hefei, Anhui, 230026, P. R. China, [2]iChEM, University of Science and Technology of China, 96 Jin Zhai Rd, Hefei, Anhui, 230026, P. R. China

1. Characterizations

Scanning electron microscopy (SEM) imageswere taken with a JSM-6700F (JEOL, Japan). Atomic force microscopy (AFM) images were taken with a DI Innova (Veeco, USA) and the radius of tip (RTESPA: MPP-11120-10, Bruker company, German) is ~ 8 nm. Transmission electron microscopy (TEM) imageswere taken with a JEM-2010F and HRTEM images were taken with a JEM-ARM200F (JEOL, Japan) packaged with a STEM Cs-corrector and operated at 200 kV. Raman spectrum was measured using a Labram-HR (JY, France) with laser excitation at 532 nm. Fourier transform infrared spectroscopy (FTIR) spectrum was measured using a Nicolet 8700 (Thermo Scientific Instrument, USA). X-ray photoelectron spectroscopy (XPS) spectrum was measured using an Escalab 250 (Thermo-VG Scientific). X-ray diffraction spectrum was measured using a SmartLab (Rigaku, Japan). Element analysis was performed using a Vario EL Cude (Elementar, German) and the combustion temperatures were 950 and 1150 ^{o}C for C/H and O, respectively. UV-vis-NIR absorption spectra were obtained using a DUV-3700 (Shimadzu, Japan). Photoluminescence (PL) spectrum and PL lifetime were recorded using a Fluorolog-3-TAU (Jobin Yvon, France).

2. Bioimaging

Cell imaging was examined using a confocal laser scanning microscope (CLSM) with HepG2 cells, representative mammalian cells. Dulbecco's Modified Eagle's Medium (DMEM) high glucose supplemented with 10% (v/v) heat inactivated fetal bovine serum (FBS) was used as cell culture media, and HepG2 cells were grown at

37°C under conditions of 5% CO_2. Approximately 50,000 cells were seeded on a confocal coverslip and cultured overnight. After that, the cells were washed with DMEM free-FBS culture and replaced with as-prepared CQDs of 200 μg mL^{-1} in DMEM or DMEM as a control for 2 h at 37 °C. Subsequently, the supernatant was carefully removed and the cells were washed three times with 1×PBS (7.4). The resultant cells were re-suspended in 1×PBS (7.4) and imaged under a CLSM imaging system with 63×oil lens. Laser lines of 405 at approximately 80% of their maximum intensity were used to excite CQDs. Controls are those assayed similarly but without CQD addition. As shown in Figure S2, the CQDs could produce photoluminescence when excited with 405 nm, and mainly occurred in the cytoplasm and cytoderm, while others don't. This suggested that CQDs uptake into mammalian cells could be used for bioimaging.

3. Supercapacitor measurement

A two-electrode cell device was used to measure the performance of supercapacitors with annealed CQD assembly ($CQDs_{-800}$ and $CQDs_{-600}$). 5wt.% Polytetrafluoroet-hylene (PTFE; 5wt.% dispersion in water) was added to the annealed CQD assembly powder as a binder, and 10wt.% carbon black (CB) was added as conductive additive.Typically, the annealed CQD assembly, PTFE and CB were mixed into a paste using a mortar and pestle, rolled into uniform thickness sheets whose thickness ranged 30 to 80 μm thick and punched into ~0.7 cm diameter electrodes. A pair of typical electrodes had a weight between 1.8 and 2.5 mg after drying overnight at ~ 110 °C under

vacuum. Two identical (by weight and size) electrodes were assembled in a test cell as shown in Ref [1], which consisted of two current collectors, two electrodes, and an ion-porous separator (Celgard® 3501) supported in a test fixture consisting of two stainless steel plates. Pt foils were used as current collectors. 6 M KOH water solution was used as electrolyte.

The gravimetric and volumetric capacitances of one electrode from galvanostatic discharge curves were calculated by using the formula [2, 3]:

$$C_{gra} = \frac{2I\Delta t}{m\Delta V}$$

$$C_{Vol} = \rho \times C_{gra}$$

Where C_{gra} is the gravimetric capacitance in a single electrode, C_{Vol} is the volumetric capacitance in a single electrode, I is the constant current, Δt is the discharge time, m is the mass for one electrode, ΔV is the potential window during the discharge process, and ρ is the density of the electrode.

4. Figures and Tables

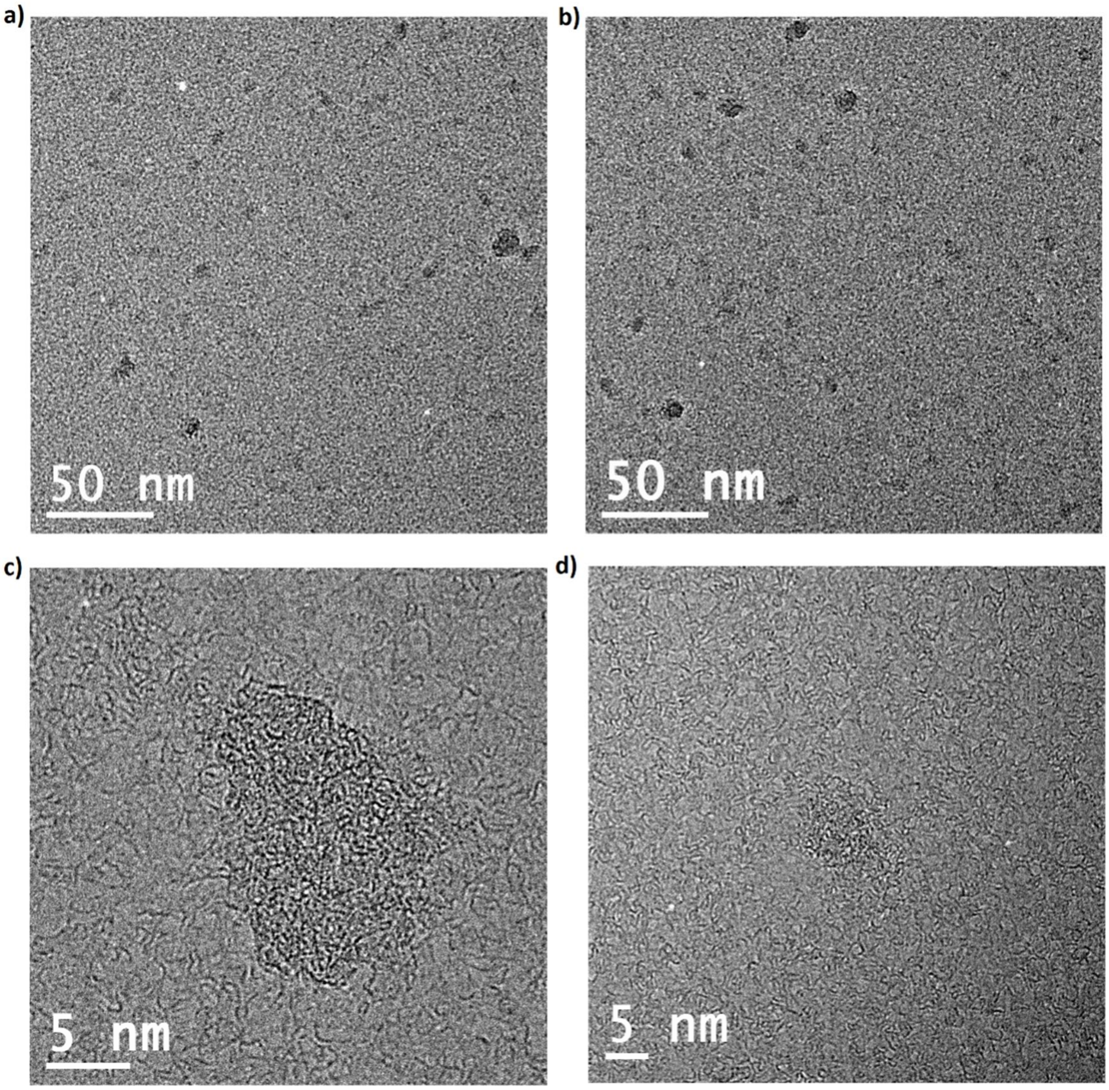

Figure S1.(a) and (b) are TEM images of CQDs. (c) and (d) are HRTEM of two isolated CQDs.

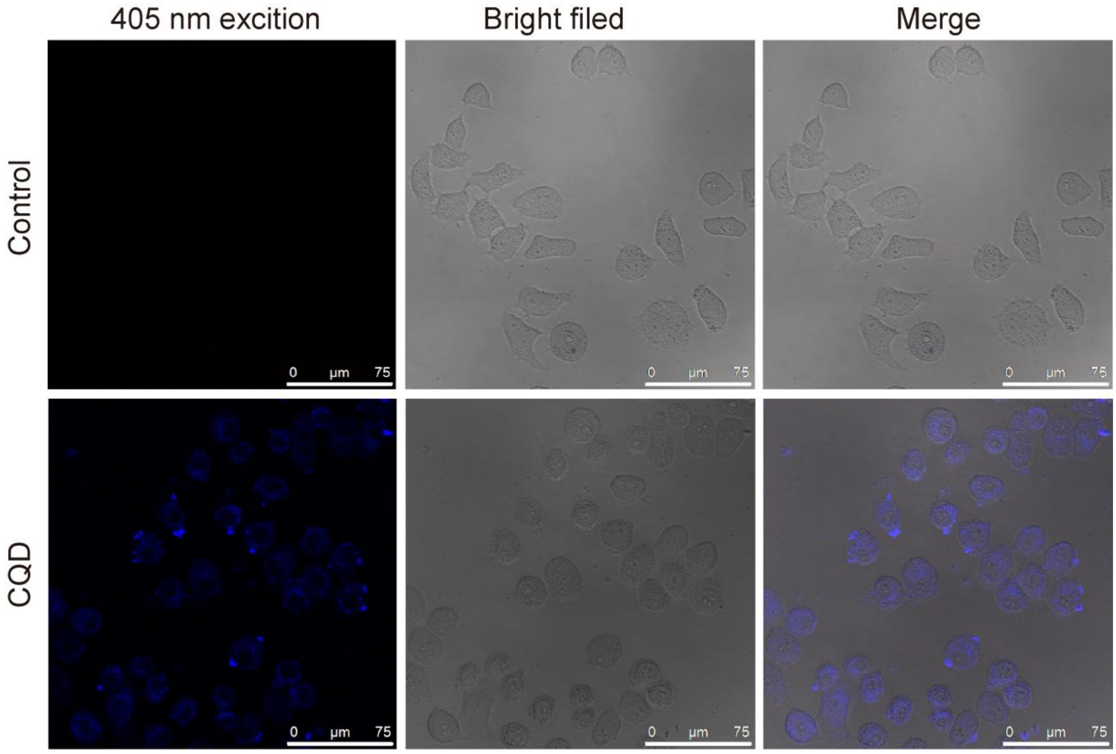

Figure S2. Fluorescence confocal microscopy images of HepG 2 cells treated with as-prepared CQD suspension (0.2 mg/mL in serum-absent DMEM medium) for 2 h. Cells assayed similarly but without CQD addition are included as control.

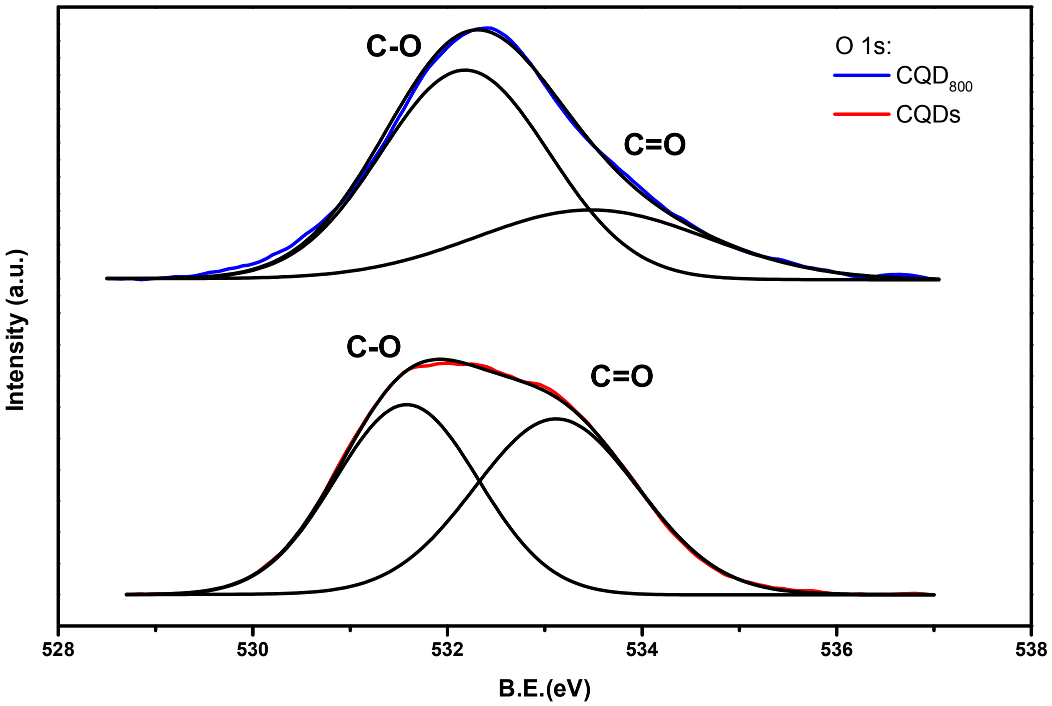

Figure S3. O 1s XPS of CQD assembly and CQD_{-800}.

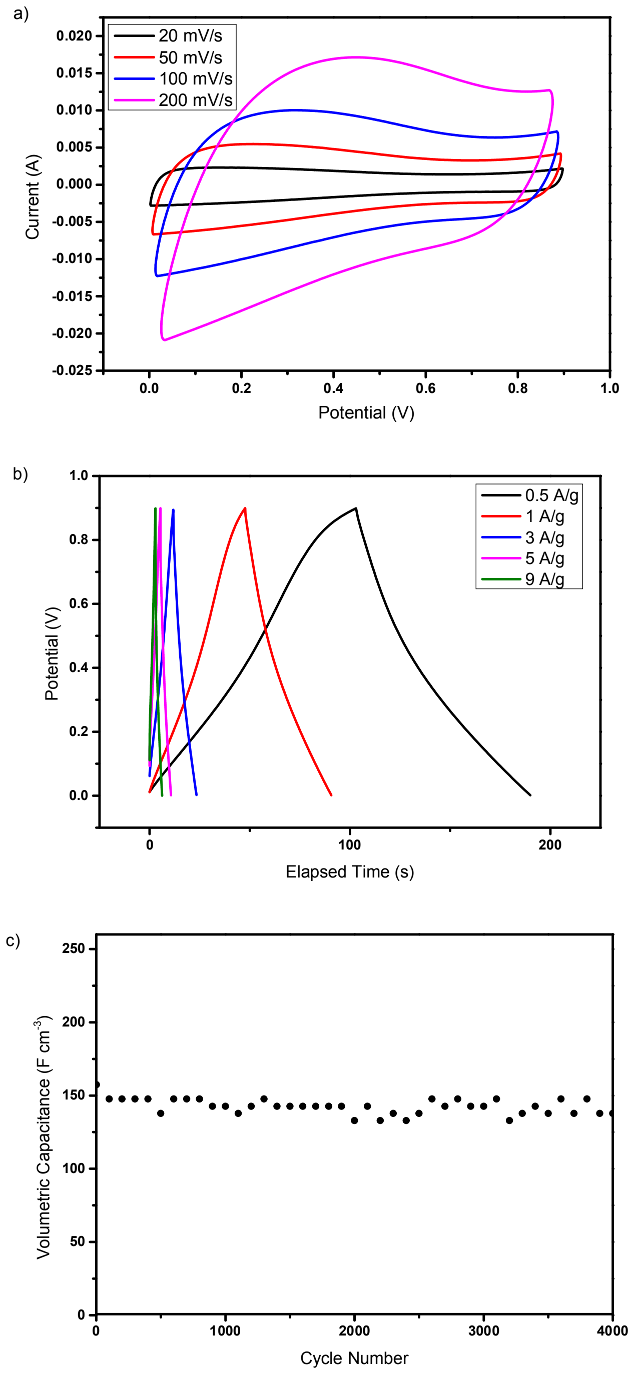

Figure S4. (a)CV, (b) galvanostatic charge/discharge curves and (c) 4000 charge/discharge cycles at 2 A g^{-1} for a CQD_{-600} based supercapacitor.

Table S1. Volumetric capacitances of various carbon materials aqueous electrolytes

Material	Electrolyte	Density (g cm^{-3})	C_v (F cm-3)	Test device	Ref.
HPGM	6 M KOH	1.58	376	Two-electrode	3
PVFC700	6 M KOH	0.826	218	Two-electrode	4
RGO paper	6 M KOH	0.94	196	Three-electrode	5
AC500	6 M KOH	0.504	171	Two-electrode	6
CXAG-30	1 M H_2SO_4	0.8	166	Three-electrode	7
**CQDs$_{-600}$**	**6 M KOH**	**1.23**	**154**	**Two-electrode**	**Here**
TiC-CDC	1 M H_2SO_4	-	140	Two-electrode	8
GPCP-900s	1 M H_2SO_4	0.58	135	Three-electrode	9
LBL-MWCNT	1 M H_2SO_4	-	132	-	10
PGNs	6 M KOH	-	101	Three-electrode	11
Carbon cloth	KOH	0.35	70	-	12

5. References

[1] M. D. Stoller, *et al.* Energ Environ Sci 3 (2010),1294-1301.
[2] X. W. Yang, *et al.* Science 341 (2013),534-537.
[3] Y. Tao, *et al.* Sci Rep-Uk 3:2975 (2013).
[4] B. Xu, *et al.* J. Power. Sources 228 (2013), 193-197.
[5] Z. Lei, *et al.* Energy Environ. Sci. 5 (2012), 6391-6399.
[6] B. Xu, *et al.* Mater. Chem. Phys. 124 (2010), 504-509.
[7] Z. Zapata-Benabithe, *et al.* Langmuir 29 (2013), 6166-6173.
[8] J. Chmiola, *et al.* J. Power. Sources 158 (2006), 765-772.
[9] D. -W. Wang, *et al.* ACS Nano 3 (2009), 1745-1752.
[10] S. W. Lee, *et al.* J. Am. Chem. Soc. 131 (2008), 671-679.
[11] Z. Fan, *et al.* Carbon 50 (2012), 1699-1703.
[12] P. Simon, *et al.* The Electrochemical Society Interface (2008), 38-43.
